# Women with Fibromyalgia Prefer Resistance Exercise with Heavy Loads—A Randomized Crossover Pilot Study

**DOI:** 10.3390/ijerph18126276

**Published:** 2021-06-10

**Authors:** Ulf Mathias Andersson, Anna Cristina Åberg, Lena von Koch, Annie Palstam

**Affiliations:** 1 School of Health and Welfare, Medical Science, Dalarna University, 791 88 Falun, Sweden; aab@du.se (A.C.Å.); apl@du.se (A.P.); 2 Smärtmottagningen Falun, Dalarna Region, 791 31 Falun, Sweden; 3 Smärtehabilitering Säter, Dalarna Region, 783 32 Säter, Sweden; 4 Department of Public Health and Caring Sciences, Uppsala University, 751 85 Uppsala, Sweden; 5 Department of Neurobiology, Care Sciences and Society, Karolinska Institutet, 171 76 Stockholm, Sweden; lena.von.koch@ki.se; 6 Institute of Neuroscience and Physiology, Sahlgrenska Academy, University of Gothenburg, 413 45 Gothenburg, Sweden; 7 Department of Rehabilitation Medicine, Sahlgrenska University Hospital, 413 45 Gothenburg, Sweden

**Keywords:** chronic widespread pain, physical, training, lactate, rehabilitation

## Abstract

Fibromyalgia (FM) is a chronic pain condition associated with impaired muscle strength and exercise-induced pain. Physical exercise has been highlighted, by international clinical guidelines and stakeholders, as an essential component of rehabilitation in FM. Exposure to pain during exercise is generally correlated with elevated lactate levels and, additionally, is one known reason for persons with FM to avoid physical exercise and activity. A crossover design was used to test and evaluate an approach consisting of resistance exercise with heavy loads and a low number of repetitions among ten women with FM. The participants were consecutively recruited to test and perform exercise with two different resistance levels (A = light/moderate load, and B = heavy load) in a randomized crossover trial using an AB/BA setting. Results showed that the heavy load exercise session was experienced as more positive than the light/moderate load exercise session and that lower lactate levels followed exercise with heavier weight loads. This is promising and indicates that the approach of heavy weight loads and accustomed repetitions is accepted in FM and has the potential to attenuate hesitation to exercise due to exercise-induced pain. However, these effects need to be further investigated in more extensive studies.

## 1. Introduction

Fibromyalgia (FM) is a chronic pain condition, characterized by persistent widespread pain and increased pain sensitivity [1], associated with fatigue [2] and considerably impaired muscle strength [3,4], which affects daily life [5,6]. FM is classified according to the 1990 American College of Rheumatology (ACR) classification criteria [1] as including widespread pain and tenderness in >11 of 18 tender-point sites and has a global prevalence of around 2% [7]—being more common among women [8]. The pathophysiological mechanisms in FM involve peripheral tissues as well as the central nervous system, although yet to be fully clarified [9,10]. Numerous somatic, psychological, and social factors are associated with FM [11,12,13,14], including neuroinflammation and chronic systemic inflammation [15]. There is a parallel dysfunction in pain regulation and the endogenous pain inhibitory systems [16] normally resulting in exercise-induced pain [17]. Such activity-related symptoms correlate with elevated lactate levels [9,18,19].

Physical exercise has been highlighted, by international clinical guidelines and stakeholders, as an essential component of rehabilitation in FM [20], as physical exercise has been shown to improve physical function, reduce pain and enhance quality of life in persons with FM [20]. Additionally, it has been shown that women with FM have the ability to increase muscle strength by resistance exercise similar to healthy women [21] and that resistance exercise with high intensity is safe for FM to perform [20,22,23].

Despite this, persons with FM generally prefer to exercise with light/moderate loads [24,25] and with a slow progression, due to commonly occurring exercise-induced pain [9,21,26,27,28]. This approach is adopted in clinical recommendations [29]. Hence, persons with FM are rarely introduced to alternative resistance exercise approaches. As a result, progression of loading is often slow in rehabilitation settings [21,26,27,28]. Accordingly, persons with FM only gain modest treatment effects of resistance exercise interventions and fail to reach sufficient levels of muscle activation [20] to attain optimal endogen release of importance on systemic inflammation [30]. Furthermore, the exposure to pain during exercise is a known reason to avoid physical activity per se [31], which forces persons with FM to lead a sedentary lifestyle [32]. In turn, this often leads to inactivity-induced consequences in a ‘vicious circle’ where lower muscle strength is associated with higher levels of pain in FM [33].

So far, an unexplored way to conduct rehabilitation for persons with FM may be based on an approach consisting of resistance exercise starting with heavy loads but with only a very low number of repetitions, which is already well established and frequently used in the context of athletic performance in sports [34]. A high-intensity resistance exercise over a short period of time mainly achieves energy providence through anaerobic systems, which does not generate considerable lactate production due to the available creatine phosphate in the working muscle [35].

The hypothesis was that heavy load resistance exercise could be well accepted in women with FM when conducted with few repetitions, which was based on an attempt to take physiological stress into account. We therefore conducted this pilot study with the aim to compare how women with FM perceived a heavy load resistance exercise session versus a light/moderate load resistance exercise session and to evaluate lactate levels after performing each resistance exercise session. Our intention was also to evaluate any associations between experiences of the resistance exercise session and measured lactate levels. This pilot study was carried out to provide a basis for further, more extensive research.

## 2. Materials and Methods

A randomized crossover trial was used in an AB/BA setting [36], with a one-to-two-weeks washout between the visits, enabling the participants to relate to two sessions with different loads of resistance exercise. Ten women with FM (diagnosed using the ACR 1990 criteria) undergoing rehabilitation at a Regional Interdisciplinary Pain Rehabilitation Clinic in middle Sweden, specifically focusing on rehabilitating and diagnosing patients with different complex chronic pain conditions, were recruited for the study. Inclusion criteria: premenopausal women diagnosed with FM, 18–50 years of age, regardless of previous experience in resistance exercise. Exclusion criteria: being a smoker, experience of any spinal surgery, severe anxiety or depression or other comorbidity impeding performing resistance exercise. Participants were consecutively included until ten had entered the study.

The procedure consisted of three visits, the first visit to test the participants’ strength, and the second and third visit to perform different resistance levels (Table 1). At the first visit, before any measurements, the participants were asked to mark their current pain intensity on a 100 mm ungraded visual analogue scale (VAS) [37], and history of pain was also reported. Then, they performed a five-minute warm-up on a cycle ergometer reaching 110–130 heartbeats/minute, followed by an introduction to six resistance exercises targeting major muscle groups: (1) bench press (conducted on a Smith machine); (2) shoulder shrug with barbell to chin (conducted on a Smith machine); (3a) barbell lunge, left leg; (3b) barbell lunge, right leg; (4) latissimus pull down with a supinated grip (conducted on a cable machine); (5a) bicep curl on a Scott bench, left arm; (5b) bicep curl on a Scott bench, right arm; (6) squats on a flat weight bench (conducted on a Smith machine). Furthermore, a one Repetition Maximum (1RM) [38] was instructed and tested out on the six resistance exercises, respectively (together with a physiotherapist experienced in pain rehabilitation). This was conducted after instruction and a warm-up where the participants performed the exercises with double attempts on a gradually increasing load until failure (i.e., inability to perform the exercise due to exhaustion, aggravated pain or due to severe compensatory movements). Based on individually adjusted starting loads, a 5 kg interval was used to increase the weight load in all resistance exercises exceeding 20 kg (all exercises except the bicep curl). In the bicep curl, a 1–2 kg interval was used for load progression. 

A one-minute rest between the lifts was applied. All participants reached failure within five attempts, and the highest weight load achieved was referred to as 1RM. A two-minute rest was used between each resistance exercise, respectively.

When arriving at the clinic on their second visit, approximately one to two weeks after the first visit, a computer-generated randomization for group affiliation [39] was carried out. This was guided by a study administrator blinded for participants’ group allocation to randomly assign the participants to start the resistance exercise session with different loads [40], on either (A) 50% [41,42] or (B) 80% [42,43] of 1RM. The result of the randomization was reported by the study administrator to the responsible physiotherapist and the patient at the same occasion, before starting the session.

When resistance exercise was performed with 50% of 1RM, the participants were expected to manage 20–30 repetitions [44,45] in no more than 60–90 seconds, and when performed with 80% of 1RM, 7–8 repetitions [44,45] in no more than 12–15 s. The resistance exercises were performed according to instructions from the supervising physiotherapist, and the participants were asked to correct the motion if severe compensatory movements were observed. When reaching either the maximum number of repetitions or seconds, or if the motion differed considerably from the intended motion, the participants were requested to abort the set. The six different resistance exercises were performed in one set until maximum repetitions or maximum seconds were reached. A two-minute rest was conducted between the six different resistance exercises.

Two minutes after the performance of the resistance exercise session, blood lactate levels were analyzed using the Accutrend^®^ Plus meter (Roche, Rotkreuz, Switzerland) with semi-capillary blood taken from the side of the fingertip. While waiting for the analysis result, the participants were asked (by the physiotherapist experienced in pain rehabilitation) to report “What is your experience of the training session right now?”. Four alternative answers were possible: (1) Very negative; (2) Negative; (3) Positive; (4) Very positive. The session ended with a five-minute cool down on the cycle ergometer.

At the third visit, after a wash-out period of one to two weeks, the participants performed the resistance exercise session, just as in the second visit, although this time at the other intensity level (Table 1).

### Statistical Analyses

IBM SPSS Statistics 25 (IBM, Armonk, NY, USA) was used to manage and analyze data. Descriptive results are presented as median and inter quartile range (IQR), or frequencies. Statistical analyses were performed using non-parametric statistics given the small sample size. Analyses of differences between the heavy and light/moderate load resistance exercise sessions were conducted using the Wilcoxon Matched-Pairs Signed-Rank Test. Spearman rank correlation (rs) was used to analyze correlations between lactate levels and experiences of the sessions. The significance level was set at *p* < 0.05.

## 3. Results

Descriptive information of study participants, including reported pain before the resistance exercises were initiated, is reported in Table 2. Participants reported a history of pain with onset of 160 months (12–385 months) and had an FM diagnosis since 12 months (1–45 months).

None of the participants stated previous experience of heavy load resistance exercise, and the median 1RM on the six resistance exercises was: (1) bench press: 25 (range = 15–40) kg; (2) shoulder shrug: 25 (12.5–30) kg; (3a) barbell lunge, left: 16 (8–35) kg; (3b) barbell lunge, right: 16 (6–35) kg; (4) latissimus pull down: 80 (50–100) kg; (5a) bicep curl, left: 6.5 (4–10) kg; (5b) bicep curl, right: 7 (4–10) kg; (6) squats: 30 (2.5–35) kg.

**Figure 1:**
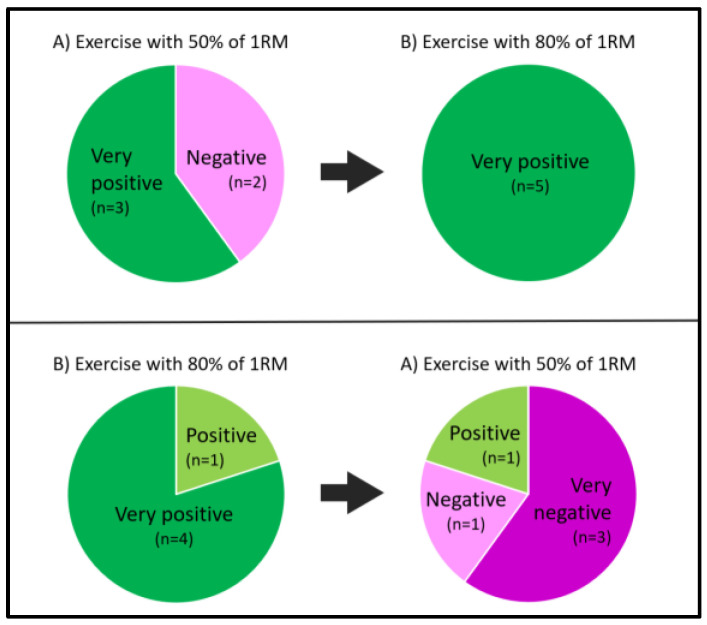
Participants’ experiences and group differences of the resistance exercise session starting with (**A**) 50% of 1RM followed by (**B**) 80% of 1RM versus starting with (**B**) 80% of 1RM followed by (**A**) 50% of 1RM. (1RM = One Repetition Maximum).

The heavy load resistance exercise session (80% of 1RM) was perceived to be more positive (*p* = 0.016) than the light/moderate resistance exercise session (50% of 1RM) (Figure 1). The participants performed an average of 22 repetitions in exercises with light/moderate loads (50% of 1RM) and an average of six repetitions in exercises with heavy loads (80% of 1RM). Full exercise compliance was achieved during performance of the exercises with heavy loads (80% of 1RM), but one participant declined the exercises: (4) latissimus pulldown and (5) bicep curl, during the exercise session with light/moderate resistance exercise loads (50% of 1RM) due to stated discomfort. It is to be noted that the weight load and repetitions were expected to be similar regarding muscle effort in a strength testing setting, although the total exercise time and lifted load volume were markedly shorter/lower in the session using 80% of 1RM.

The between-group comparison showed significantly more negative experience when performing the light/moderate resistance exercise session after starting with the exercise of heavy loads as compared to the group starting with the light/moderate resistance exercise session (*p* = 0.008) (Figure 1).

**Figure 2:**
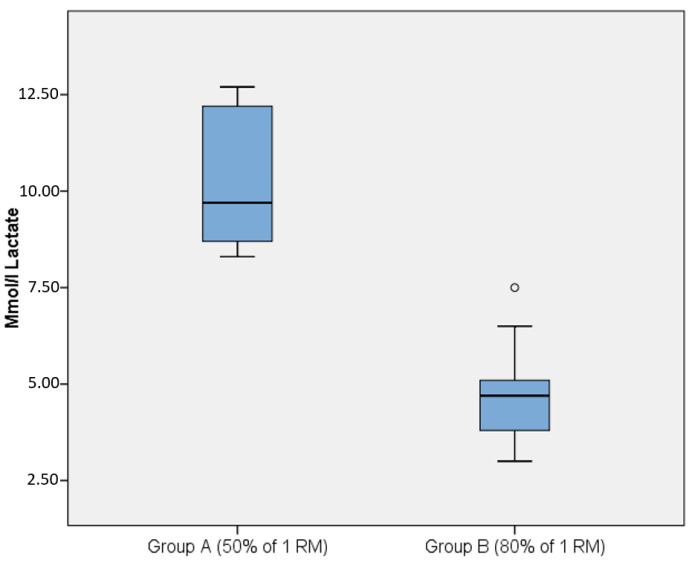
Lactate levels in mmol/l after completed resistance exercise for the group using (**A**) 50% of 1RM and for (**B**) 80% of 1RM. An outlier in Group B (80% of 1RM) is marked with a circle.

The lactate levels after performance of the light/moderate load (50% of 1RM) resistance exercise session were significantly higher (*p* = 0.005) than after performance of the heavy load resistance exercise session (80% of 1RM) (Figure 2). We found no significant correlation between the estimated experiences of the sessions and lactate levels (rs = 0.39, *p* = 0.09).

**Figure 3:**
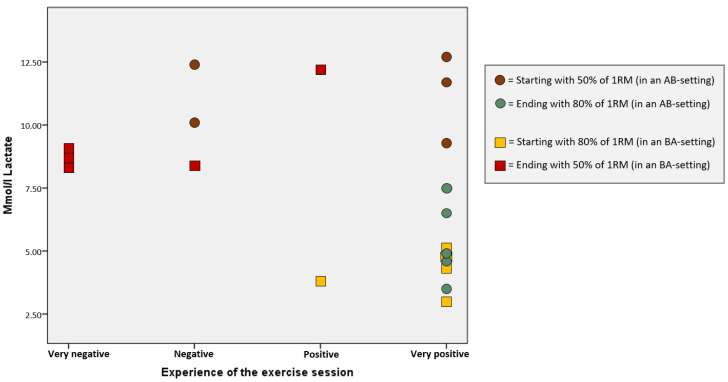
Scatter plot of lactate levels and participants’ experiences after the resistance exercise sessions.

No significant correlation was shown between lactate levels and experiences in our results. Still, a pattern of negative correlation between lactate levels and participants’ experiences of the resistance exercise session was indicated by a scatterplot (Figure 3).

## 4. Discussion

Participants preferred the heavy load resistance exercise session using 80% of 1RM, which was experienced as more positive than the light/moderate resistance exercise session using 50% of 1RM. Additionally, lactate levels were shown to be significantly lower (*p* = 0.005) when the women with FM executed a heavy load resistance exercise session of 80% of 1RM compared to 50% of 1RM. This is presumably due to sufficient levels of creatine phosphate [35] in the working muscle during a limited period of muscle activation and a lower lifted load volume.

The results indicated a pattern of negative correlation between lactate levels and participants’ experiences of the resistance exercise session (Figure 3), in line with previous findings of positive correlation between ratings of perceived exhaustion and lactate [46]. Other studies show higher concentrations of lactate in women with FM, both at rest [47] and after exercise [48]. However, the role of lactate, as well as other variables related to intense physical activity, is still intriguing [49]. Exercise-induced pain [9], in addition to high lactate levels [19], might be a reason to why women with FM prefer exercises generating lower ratings of perceived exhaustion [24,46] even though the relationship between physical activity and central pain processing is less clear in patient populations [50].

Our suggested heavy load resistance exercise approach with a low number of repetitions and a lower lifted load volume may circumvent accumulation of lactate, and hence enable an exercise setting with tailored muscle stimulation [40]. Recent studies show that skeletal muscles work as an endocrine organ, which can produce and secrete muscle related cytokines (myokines) in response to high-intensity exercise [30,51,52,53]. These findings may be relevant for rehabilitation in FM since myokines can hold an anti-inflammatory effect, which is of special importance for systemic inflammation [53]. Hence, a well-accepted heavy load exercise intervention might, therefore, affect muscle stimuli beneficial to gain muscle mass and, as a result, increase muscle-induced endocrine regulation [51,52]. Improved understanding of peripheral muscle alterations and their relevance on aspects of pain and systemic inflammation [47] may lead to future modified interventions in rehabilitation for women with FM. An interesting, unexpected observation was that none of the women with FM spontaneously reported augmented pain during the 1RM-test, nor during the 80% of 1RM resistance exercise session, but pain was commonly reported during the 50% of 1RM resistance exercise session.

The used AB/BA cross-over study design is well suited to chronic diseases and conditions, such as FM, given the relative consistency of physical functioning over time. However, it is crucial that participants perform both interventions in an AB/BA setting, and, in this pilot study, all participants fulfilled all sessions and related assessments. The study demonstrates statistically significant differences between interventions, despite a small sample size. However, no significant correlation was found between lactate levels and exercise experiences, which may be due to the small sample size and possible ceiling effect of the experience ratings. Data from this study may be used as a basis for sample size calculation in a main study with similar arrangements, where a matching load volume also may be considered. However, it may be speculated that the heavy load resistance exercise is more vital [40] than the specific exercises used in this pilot study.

## 5. Conclusions

Potentially, our proposed strength exercise approach may yield increased exercise effects and decrease hesitation to exercise due to reduced exercise-induced pain. In addition, permitting a person with FM to perform a heavy load resistance exercise may well result in a wider psychological and physiological effect, which might affect exercise self-efficacy. However, the results of this heavy load exercise intervention with accustomed repetitions need further investigation to determine if this approach could circumvent exercise-related obstacles in settings for rehabilitation in FM.

## Figures and Tables

**Table 1:** Overview of the study procedures, including exercise sessions (A) 50% of 1RM and (B) 80% of 1RM.

First Visit	Second Visit	Washout Period	Third Visit
Testing of 1RM in six resistance exercises.	(A) Resistance exercise performed with 50% of 1RM.	Approximately one to two weeks.	(B) Resistance exercise performed with 80 % of 1RM.
	(B) Resistance exercise performed with 80% of 1RM.		(A) Resistance exercise performed with 50 % of 1RM.

**Table 2:** Descriptive information of study participants.

Characteristics	Median	Range
Age (years)	40.5	(22–46)
Height (m)	1.68	(1.58–1.79)
Weight (kg)	84.4	(62–95)
Body Mass Index (kg/m^2^)	28.1	(22.2–35.3)
Current pain, VAS (0–100 mm)	58.5	(29–87)

## Data Availability

The material analyzed during the current study is not publicly available due to its content of sensitive personal data. Datasets generated may be available from the first author (M.A.) on reasonable request, after ethical considerations.
